# Advanced Diagnosis of Hypertrophic Cardiomyopathy with AI-ECG and Differences Based on Ethnicity and HCM Subtype

**DOI:** 10.3390/jcm14134718

**Published:** 2025-07-03

**Authors:** Myra Lewontin, Emily Kaplan, Kenneth C. Bilchick, Anita Barber, Derek Bivona, Christopher M. Kramer, Anna Parrish, Karen McClean, Matthew Thomas, Allison Perry, Kaitlyn Amos, Michael Ayers

**Affiliations:** Cardiovascular Division, Department of Medicine, University of Virginia Health System, Charlottesville, VA 22903, USA; uxf6nf@virginia.edu (E.K.); kcb7f@uvahealth.org (K.C.B.); arb4p@uvahealth.org (A.B.); djb6ab@virginia.edu (D.B.); cmk2n@uvahealth.org (C.M.K.); aes4b@uvahealth.org (A.P.); km2yr@uvahealth.org (K.M.); mjt6e@uvahealth.org (M.T.); qry6nr@uvahealth.org (A.P.); kka5d@uvahealth.org (K.A.); mpa2h@uvahealth.org (M.A.)

**Keywords:** hypertrophic cardiomyopathy, magnetic resonance imaging, electrocardiogram, artificial intelligence

## Abstract

**Background/Objective:** Hypertrophic cardiomyopathy (HCM) often presents later in the disease course, with frequent misdiagnoses and population-level underdiagnoses. Underserved patients may have even greater diagnostic delays. We aimed to test the hypothesis in a retrospective cohort that artificial intelligence analysis of ECGs (AI-ECG) could have afforded the opportunity for earlier diagnosis of HCM in one health system. **Methods:** We collected all available ECGs from patients referred to an HCM Center of Excellence over 15 years, both before and after HCM diagnosis. We applied AI-ECG to each ECG in a blinded fashion to predict the probability of HCM. We calculated the time between each patient’s AI-ECG diagnosis and clinical diagnosis. We examined the sensitivity and specificity of AI-ECG for all patients, and by septal subtype and genetic test result. **Results:** 3499 ECGs were analyzed in 404 patients (age 56 ± 18 years, 52% female). AI-ECG correctly identified HCM in 155 patients with a sensitivity of 67%, specificity of 95%, positive predictive value of 94%, and a negative predictive value of 69%. The AUC was similar using mean probability from all ECGs for each patient (AUC 0.91 [0.88, 0.94]) or using probability from the first ECG (AUC 0.91 [0.87,0.93]). AI-ECG diagnosed 27 patients over 1 year before clinical diagnosis, and up to 16.3 years early. Black patients were more likely than White patients to have an AI-ECG diagnosis before a clinical diagnosis (*p* = 0.005). **Conclusions:** AI-ECG offers the potential for advanced HCM diagnosis. Differences in identification timing between subgroups highlight inequities in current care and show the potential of AI-ECG for the greatest benefit in underserved ethnic groups.

## 1. Introduction

Hypertrophic cardiomyopathy (HCM) is often misdiagnosed or has significant delays to diagnosis. Population studies suggest the condition affects an estimated 1:200–1:500 people [1], though ICD10 data suggest only 1:3000 patients have received a clinical HCM diagnosis [2,3,4]. In one study, over half the included patients had been previously misdiagnosed with another condition or otherwise had a delay in HCM diagnosis. These delays were associated with economic and clinical consequences [5,6]. Additionally, HCM patients are frequently only referred for diagnostic testing following significant symptom onset or an acute cardiac event [7]. Ideally, asymptomatic individuals would receive appropriate risk stratification prior to an acute event. Timely diagnosis of HCM allows for the initiation of medical and/or septal reduction therapy to address symptoms and improve quality of life, appropriate risk stratification for sudden cardiac death with potential primary prevention via ICDs [8], and screening for and treatment of comorbidities such as atrial fibrillation which result in high rates of stroke for patients with HCM [9,10,11].

The diagnosis of HCM is based on maximal wall thickness and measured on echocardiogram or cardiac magnetic resonance imaging [12]. An imaging-based diagnosis requires access to both imaging facilities and a specialist for interpretation. This can create delays in diagnosis, particularly for patients with geographic or economic barriers to accessing care. While not diagnostic, electrocardiograms (ECG) can play a role in screening for HCM among other cardiac conditions. The 12-lead ECG is a quick, inexpensive, and commonly used screening test that can be performed in a wide variety of medical settings. An estimated 90–96% of HCM patients have ECG abnormalities including P-wave prolongation, increased QRS complex voltage, and repolarization abnormalities [13,14,15,16,17,18,19]. However, these ECG patterns are nonspecific, potentially leading to high numbers of false positives using ECG screening.

Artificial intelligence-based detection of HCM from ECGs has emerged as a potential approach to incorporating ECG analysis into HCM diagnosis [20]. Recent models have used convolutional neural networks to identify HCM, although with high false positive rates, particularly in older patients [21,22,23,24]. Currently, the clinical utility of artificial intelligence (AI) in the ECG-based diagnosis of HCM is uncertain, but an appropriately designed model has the potential to accelerate diagnosis and treatment to improve clinical outcomes. This study aims to evaluate the yield of an AI-based algorithm for identification of potential HCM cases from analysis of 12-lead ECGs and to examine the timing of potential AI identification relative to clinical diagnosis.

## 2. Materials and Methods

The study was approved by the University of Virginia Institutional Review Board for Human Subjects Research. Research was conducted according to the principles outlined in the Declaration of Helsinki.

We performed a blinded retrospective analysis of an AI algorithm (Viz.ai) to predict HCM from serial 12-lead ECGs. All ECGs available for each patient in the medical record were evaluated. We considered an alternative methodology of assigning ECG findings to uniform time intervals relative to the time of diagnosis but opted for the more conservative approach of using the actual times, as the former approach could have made the lead time even greater. The method offers an end-to-end approach for detecting suspected HCM and directing patients for further evaluation. Viz.ai analyzes all 12-lead ECGs throughout a health system to flag and triage suspected HCM cases to HCM specialists for diagnostic workup. The algorithm was developed based on over 830,000 ECG exams from 300,000 individuals, including patients with obstructive and non-obstructive HCM. Derivation and validation of the algorithm included source cohorts from multiple locations globally that included racial and ethnic differences seen in PQRST wave morphologies.

Patients were selected for this study at the University of Virginia Health if they had a diagnosis of HCM at the institution’s HCM Association (HCMA) Center of Excellence (COE) or had been referred for HCM evaluation and had ECGs available before diagnosis. HCM diagnosis was made according to the American College of Cardiology/American Heart Association guidelines [12]. All included patients underwent a manual chart review to confirm their diagnoses. Patients were designated as part of the HCM group if they met HCM diagnostic criteria and as part of the control group if they did not. All ECGs available in the electronic record from before and after diagnosis by a clinician at our COE were de-identified and analyzed by Viz.ai. The Viz.ai deep neural network (DNN) for classification generates a probability for the diagnosis of HCM based on ECG features and assigns a diagnosis based on that probability. The sensitivity, specificity, positive predictive value, and negative predictive value were calculated for this Viz.ai DNN. Demographic information was obtained from the manual chart review.

Statistical analysis was performed using R version 4.2.2. Continuous variables were characterized by the mean and standard deviation or the median and interquartile range. Categorical variables were described by the number of patients and the percentage of patients in each category. Student’s *t*-tests or Analysis of Variance (ANOVA) were used for comparisons among continuous variables. Chi-square or Fisher’s exact tests were used for comparisons among categorical variables.

A chi-square test was used to compare the proportion of positive versus negative HCM predictions by the DNN in subgroups of HCM patients of interest. The potential lead time in diagnosis from the Viz.ai algorithm was calculated as the time of the first ECG with the Viz.ai prediction of HCM to the time of the actual clinical diagnosis. The date of clinician diagnosis was defined as the earliest visit date with a diagnosis of HCM listed in documentation, verified by manual chart review. The number and proportion of patients with a positive lead time, defined as ECG diagnosis prior to clinical diagnosis, was determined, and histograms demonstrating the distribution of this lead time were constructed for visualization. Lead times in patient categories of interest, including ethnicity, were compared using *t*-tests.

Logistic Regression was performed for the diagnosis of HCM based on the AI-ECG predicted probability of HCM, and receiver operating characteristic (ROC) curves were constructed using the mean of the AI-ECG predicted probabilities of all ECGs for each patient and the AI-ECG predicted probability from the earliest ECG available for each patient. ROC curves based on the earliest ECG were also constructed in subgroups of patients based on age, sex, and ethnicity. Bootstrapping was used to construct 95% confidence intervals for the areas under the curve in each ROC plot. The area under the curve (AUC) for ROC plots was compared using the DeLong method.

## 3. Results

We tested the algorithm on ECGs from 404 patients, 230 with a diagnosis of HCM and 174 controls. Demographics of the HCM and control group are listed in Table 1 and clinical characteristics of patients with HCM are listed in Table 2. Patients with HCM were, on average, older and more likely to be male.

**Figure 1:**
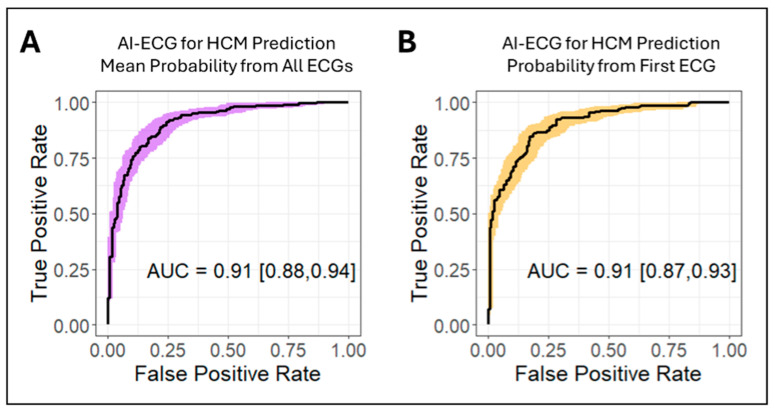
Receiver operating characteristic (ROC) plots for the detection of hypertrophic cardiomyopathy (HCM). The ROC plots are shown for the mean probability of HCM from all ECGs (**A**) and probability from the earliest ECG (**B**).

**Figure 2:**
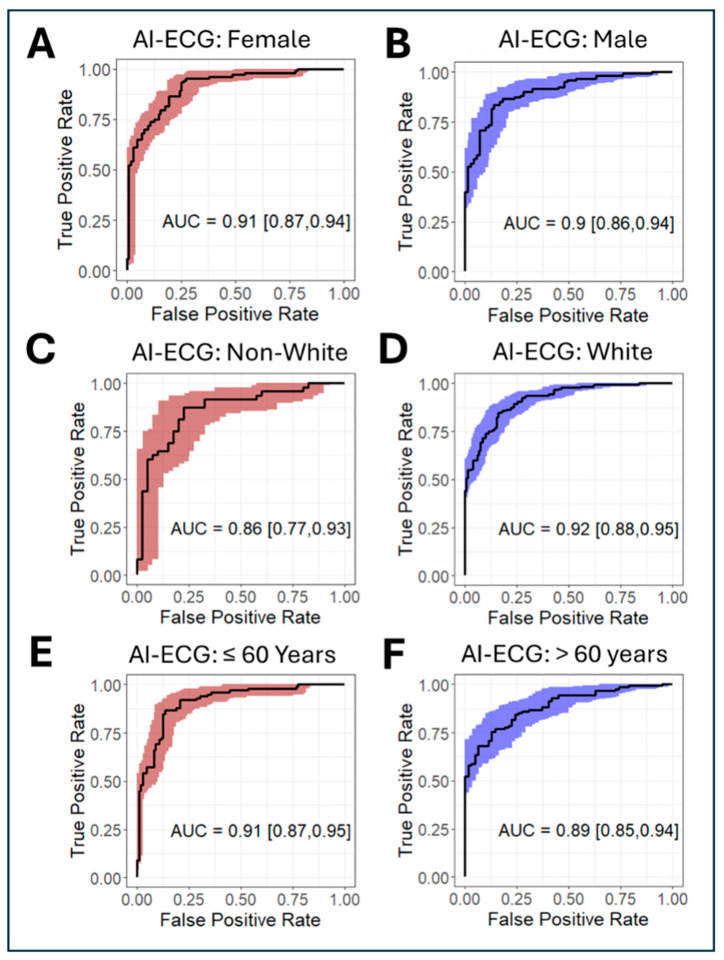
Receiver operating characteristic (ROC) plots for the detection of hypertrophic cardiomyopathy (HCM) in demographic subgroups. The ROC plots are shown for the probability from the earliest ECG in women (**A**) and men (**B**), non-white (**C**) and white individuals (**D**), and patients ≤ 60 years old (**E**) and >60 years old (**F**).

The AI-based algorithm correctly identified possible HCM in 155 patients, with a sensitivity of 67%, a specificity of 95%, a positive predictive value of 94%, and a negative predictive value of 69%. As shown in Figure 1, the AUC was similar whether the mean AI-ECG probability from all ECGs for each patient (AUC 0.91, 95% CI 0.88 to 0.94) or the AI-ECG probability from the first ECG (AUC 0.91, 95% CI 0.87 to 0.93) was used for prediction (*p* = 1). As shown in Figure 2, prediction was robust in subgroups of females (AUC 0.91, 95% CI 0.87 to 0.94) and males (AUC 0.90, 95% CI 0.86 to 0.94) (*p* = 0.97), non-white patients (AUC 0.86, 95% CI 0.77 to 0.93) and white patients (AUC 0.92, 95% CI 0.88 to 0.95) (*p* = 0.85), and patients ≤60 years old (AUC 0.91, 95% CI 0.87 to 0.95) and >60 years old (AUC 0.89, 95% CI 0.85 to 0.94) (*p* = 0.95).

HCM patients correctly identified by AI had a higher mean septal thickness than those not identified (*p* < 0.0001). The Viz.ai DNN was most accurate for patients with a left ventricular outflow tract (LVOT) obstruction versus without (*p* = 0.03), and with reverse curvature and apical patterns of hypertrophy versus other patterns of hypertrophy (*p* = 0.003). Age was similar between patients who were identified by AI and those who were not, and AI identification was not associated with sex, ethnicity, or genetic test results (Table 3).

**Figure 3:**
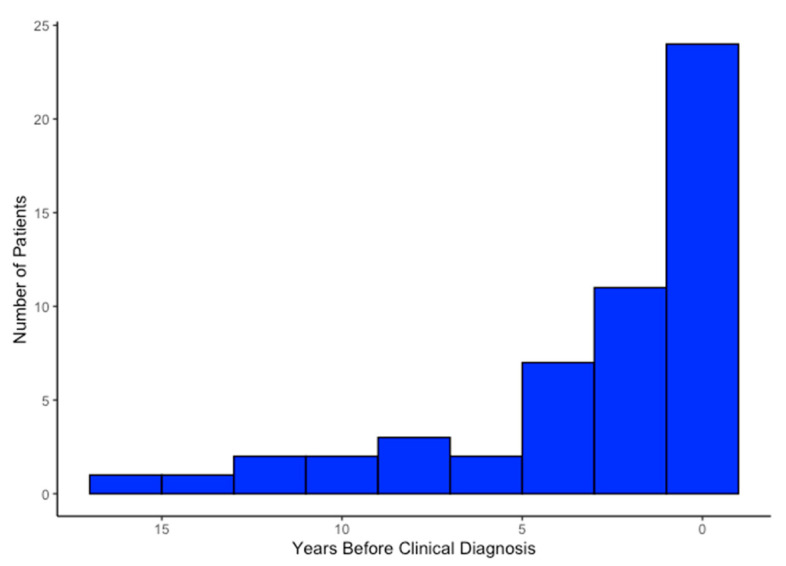
Distribution of lead times in patients with hypertrophic cardiomyopathy potentially identified by artificial intelligence prior to clinical diagnosis.

Of the patients correctly identified as having possible HCM, 53 (34%) were identified by AI from an ECG performed before clinical diagnosis. Twenty-four of these patients were identified less than a year prior to clinical diagnosis, eleven patients 1–3 years prior, seven patients 3–5 years prior, two patients 5–7 years prior, and nine patients over 7 years prior, with the longest lead time being 16.3 years prior to clinician diagnosis (Figure 3). Male sex, Black ethnicity, and the absence of LVOT obstruction were associated with AI-ECG diagnosis prior to clinician diagnosis (Table 4). Additionally, patients identified earlier by AI than by clinician diagnosis were older than those identified by AI after clinician diagnosis.

**Figure 4:**
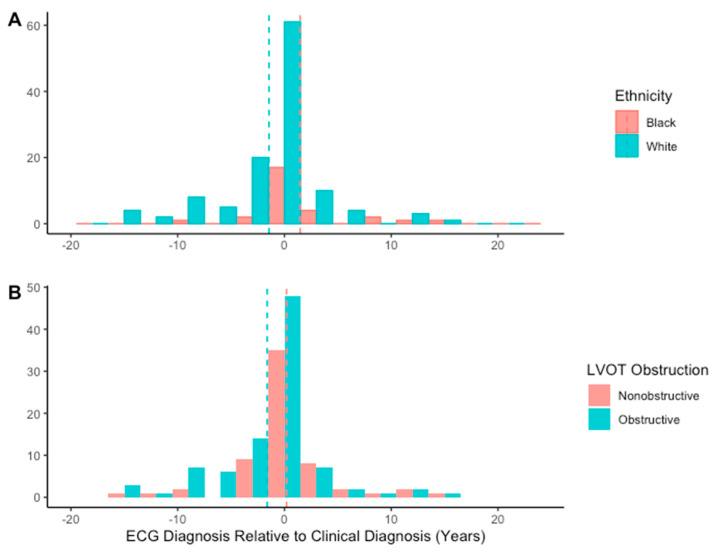
Distribution of artificial intelligence lead times in (**A**) Black and White patients; and (**B**) patients with and without a left ventricular outflow tract (LVOT) obstruction. Dashed vertical lines represent mean lead time.

Median lead time was −0.4 (IQR−27.9, 1.7) months, indicating that the first ECG identified as HCM by AI was performed 0.3 months after clinician diagnosis. Lead time for AI diagnosis did not vary significantly by sex, septal phenotype, or genetic test results (Table 5). Lead time did vary significantly by ethnicity (*p* = 0.02), however, with post hoc analysis showing a greater lead time in Black patients than in White patients (*p* = 0.005) (Figure 4). Additionally, lead time was greater in non-obstructive patients than in those with LVOT obstruction (*p* = 0.02).

At the time of diagnosis, female patients had higher BNP levels (Table 6). On cardiopulmonary exercise testing (CPET), the percent of maximal oxygen consumption (%VO_2_) at diagnosis varied by ethnicity, with post hoc testing showing that Black patients had lower %VO_2_ than White patients (*p* = 0.02). With initial cardiac magnetic resonance imaging, the late gadolinium enhancement percentage (LGE%) was higher in male patients, non-obstructive patients, and in patients with a reverse curvature septal configuration compared to those with basal septal (*p* < 0.001) or neutral (*p* = 0.002) configurations.

Eight patients without an HCM diagnosis were incorrectly identified by AI. Two of these patients had family histories of HCM and evidence of septal hypertrophy not meeting HCM criteria for maximal wall thickness, one with a pathologic variant associated with HCM and one with negative genetic testing. The remaining false positives had alternate cardiac diagnoses including left ventricular noncompaction, hypertensive cardiomyopathy, myotonia congenita, and left ventricular hypertrophy secondary to subaortic stenosis.

## 4. Discussion

The Viz.ai algorithm was able to identify HCM in over half of the patients tested. The algorithm performed particularly well in patients with larger maximal septal thickness, LVOT obstruction, and reverse curvature or apical patterns of hypertrophy. Additionally, this tool has the potential to identify HCM cases before clinician diagnosis, sometimes with significant lead time, as in the case of the patient identified from an ECG performed over 16 years prior to official diagnosis. Prior discussions of the role of AI in HCM diagnosis have been mixed, largely focusing on concerns that current tools may result in too many false positives. Our results, however, support the use of AI-ECG tools for HCM. While the instrument has imperfect sensitivity, particularly with isolated basal septal subtypes, the specificity and positive predictive values were robust (95% and 95%, respectively). Additionally, a significant number (34%) of these patients could have received an earlier diagnosis with an AI-ECG “nudge” from clinicians. In some instances, the AI-ECG tool could have potentially resulted in an earlier diagnosis by over a decade, which could have resulted in significant clinical benefit for those patients.

Also important were the variations in the lead time of AI-ECG diagnosis by demographic groups. More men were identified early by AI, although there was not a significant difference in mean lead time. This may indicate that while men were more likely to be identified early by AI, the overall difference in timing may not have clinical significance. In contrast, Black patients were identified early by AI at higher rates than White patients and had a greater lead time for AI diagnosis by almost three years. AI was equally sensitive for each ethnicity, indicating that there is likely not an intrinsic difference in analysis of the groups’ ECGs. In other words, Black patients were not more likely to be diagnosed by AI than White patients. Additionally, we have no evidence that ethnicity affects the development of ECG abnormalities in HCM, so Black and White patients should develop recognizably abnormal ECGs at the same time point in their clinical course. Given these constants, the only remaining difference is the timing of clinician diagnosis, suggesting that this difference in relative timing may represent an overall average delay in clinician diagnosis for Black patients and a pronounced disparity in care. Black patients additionally had worse exercise capacity and a trend toward higher scar burden at diagnosis, supporting the idea that they may have been diagnosed later in the disease course. If these disparities are at least in part driven by access to care, a tool such as the Viz.ai algorithm, which provides a flag for an ECG that may be obtained in a more accessible primary care setting, may help to address this by providing a greater impetus for diagnosis and escalation to specialty care.

In terms of patients’ individual physiology, non-obstructive patients could have had an earlier detection and higher lead time relative to their obstructive counterparts. This delay may correspond to differences in symptomatology or in the physical exam.

The eight false positives are also of interest. Two of them had family histories of HCM and some septal hypertrophy not meeting HCM criteria. Several others had LV hypertrophy thought to be secondary to other causes. False positive indication of HCM in these patients suggests that while they do not meet HCM guidelines, as confirmed by our HCM specialists, they may share important physiology with HCM patients. Additionally, in the cases with family histories of HCM, these may represent early phenotypes that could later develop into HCM. While our cohort lacks the number of patients needed to analyze the characteristics of our false positives, algorithms such as Viz.ai may have a role in highlighting patients that merit closer follow-up for the development of HCM.

Consideration must also be given to the implementation of a tool like this algorithm into clinical practice. AI flags like this do not replace clinical judgment, but hopefully a flag would lead to more clinical evaluation, including the discussion of symptoms, family history, and a physical exam, with the potential for referral for imaging and to specialists. Clinicians will ideally incorporate AI tools such as this one as another piece of objective data to aid in determining if further workup is appropriate and to reduce cognitive burden. As with similar tools, there is the potential for subsequent excessive use of imaging and specialty referral with the overreliance on AI, which could place stress on an already overburdened healthcare system. However, this should hopefully be mitigated by the judicious incorporation of AI-derived information, as well as the relatively high specificity and positive predictive value of this test, which increase the yield of a positive AI flag. With appropriate consideration of limitations and pitfalls, AI has been successfully incorporated into the prediction of a variety of cardiac conditions, including atrial fibrillation [25] and heart failure [26], and we posit that HCM could benefit from a similar approach. Additionally, models employing multiple machine learning modalities in an ensemble approach have shown promise in predicting cardiovascular disease [27,28], and this data piece could be incorporated into such an approach for optimal application.

Our study had a few limitations. We calculated a positive predictive value of 94% in this group of patients, although the HCM prevalence in our cohort was over half. Presumably, positive predictive value would be lower in the general population with a lower HCM prevalence, which does limit the generalizability of this tool to standard medical settings, where performance metrics would likely not be as robust. Additionally, given the retrospective nature, analysis was based on the ECGs available in electronic medical records. Therefore, ECGs were not obtained at regimented intervals, and we may not have captured the earliest time an ECG was AI-identifiable; however, this may also be considered a strength of the methodology because it represents a more conservative approach of the methodology, as additional ECGs would have the potential for an even earlier diagnosis. Finally, algorithm cutoffs were designed to maximize specificity and positive predictive value, resulting in low sensitivity and negative predictive value at 67% and 69%, respectively. Therefore, clinicians implementing the algorithm must be careful not to interpret a negative result as indicating the absence of HCM.

Despite these limitations, this study shows the ability of the Viz.ai algorithm to identify HCM cases, potentially significantly before they are clinically diagnosed. Of particular importance is the potential racial and socioeconomic ramifications of these findings. Some of the differences we have identified in relative timing of AI identification may shed light on disparities in HCM diagnosis and show a potential use of AI-ECG analysis in addressing these.

## Figures and Tables

**Table 1:** Demographics of tested patients.

		HCM	Controls	*p*-Value
*n*	404	230	174	
Age (years)	56 (18)	61 (15)	51 (18)	<0.001
**Sex**				0.01
Male	194 (48%)	123 (53%)	71 (41%)	
Female	209 (52%)	107 (46%)	103 (59%)	
**Ethnicity**				0.55
White	312 (77%)	181 (79%)	131 (75%)	
Black	68 (17%)	39 (17%)	29 (17%)	
Asian	4 (1%)	1 (0.4%)	3 (2%)	
Other	15 (4%)	9 (4%)	11 (6%)	

Data are reported as Mean (SD) or *n* (%). HCM = hypertrophic cardiomyopathy.

**Table 2:** Clinical characteristics of patients with hypertrophic cardiomyopathy.

*n*	230
Obstructive HCM	126 (55%)
Septal thickness (cm)	1.82 (0.35)
**Septal phenotype**	
Basal septal	98 (43%)
Reverse curvature	89 (39%)
Apical	23 (10%)
Neutral	16 (7%)
**Genetic test result**	
Pathogenic or likely pathogenic variant	61 (27%)
VUS	75 (33%)
Negative	77 (34%)
Not completed	17 (7%)
**NYHA at diagnosis**	
I	120 (52%)
II	70 (30%)
III	34 (15%)
IV	0 (0%)

Data are reported as Mean (SD) or *n* (%). HCM = hypertrophic cardiomyopathy, VUS = variant(s) of uncertain significance, and NYHA = New York Heart Association.

**Table 3:** Accuracy of AI identification in relevant subgroups.

	AI Positive	AI Negative	*p*-Value
Age (years)	60.9 ± 15.3	61.3 ± 15.8	0.83
**Sex**			0.18
Male	78 (63%)	45 (37%)	
Female	77 (73%)	29 (27%)	
**Ethnicity**			0.61
White	119 (66%)	62 (34%)	
Black	28 (72%)	11 (28%)	
Other	7 (78%)	2 (22%)	
Septal Thickness (cm)	1.88 ± 0.38	1.69 ± 0.26	<0.0001
**Obstructive**			0.03
Yes	93 (74%)	33 (26%)	
No	62 (60%)	42 (40%)	
**Septal Phenotype**			0.003
Basal septal	55 (56%)	43 (44%)	
Reverse Curvature	67 (75%)	22 (25%)	
Apical	21 (91%)	2 (9%)	
Neutral	11 (69%)	5 (31%)	
**Genetic Test Result**			0.13
Pathogenic or likely pathogenic variant	35 (57%)	26 (43%)	
VUS	55 (73%)	20 (27%)	
Negative	53 (69%)	24 (31%)	

Data are reported as Mean ± SD or *n* (%). VUS = variant(s) of uncertain significance.

**Table 4:** Frequency of early ECG diagnosis of hypertrophic cardiomyopathy in relevant subgroups.

	AI Identification Before Clinical Diagnosis	AI Identification After Clinical Diagnosis	*p*-Value
Age (years)	65.9 ± 12.8	58.3 ± 16.0	0.002
**Sex**			0.048
Male	33 (42%)	45 (58%)	
Female	20 (26%)	57 (74%)	
**Ethnicity**			0.005
White	34 (29%)	85 (71%)	
Black	17 (61%)	11 (39%)	
Other	2 (29%)	5 (71%)	
Septal Thickness (cm)	1.81 ± 0.33	1.91 ± 0.40	0.08
**Obstructive**			0.004
Yes	23 (25%)	70 (75%)	
No	30 (48%)	32 (52%)	
**Septal Phenotype**			0.14
Basal septal	24 (44%)	31 (56%)	
Reverse Curvature	17 (25%)	50 (75%)	
Apical	9 (43%)	12 (57%)	
Neutral	3 (38%)	8 (62%)	
**Genetic Test Result**			0.40
Pathogenic or likely pathogenic variant	9 (33%)	26 (67%)	
VUS	18 (33%)	37 (67%)	
Negative	21 (40%)	32 (60%)	

Data are reported as Mean ± SD or *n* (%). VUS = variant(s) of uncertain significance.

**Table 5:** Differences in hypertrophic cardiomyopathy diagnosis lead time in relevant subgroups.

	Lead Time (Months)	*p*-Value
**Sex**		0.87
Male	0.0 (−14.0, 3.2)	
Female	−0.9 (−37.2, 0.2)	
**Ethnicity**		0.02
White	−2.2 (−36.9, 0.5)	
Black	0.7 (−0.4, 21.4)	
Other	−2.5 (−21.1, 0.2)	
**Obstructive**		0.02
Yes	−2.5 (−38.6, 0.0)	
No	0.0 (−6.6, 11.5)	
**Septal Phenotype**		0.59
Basal septal	0.0 (−21.6, 9.6)	
Reverse Curvature	−0.9 (−24.1, 0.0)	
Apical	0.0 (−14.2, 1.1)	
Neutral	−9.3 (−51.0, 1.4)	
**Genetic Test Result**		0.66
Pathogenic or likely pathogenic variant	−2.3 (−29.9, 0.1)	
VUS	−0.2 (−11.4, 0.4)	
Negative	−0.3 (−34.6, 4.3)	

Lead time is calculated as (date of clinician diagnosis) minus (date of AI-identified ECG), with a positive lead time representing AI identification prior to clinician diagnosis. Data are reported as median (IQR). VUS = variant(s) of uncertain significance.

**Table 6:** Differences in hypertrophic cardiomyopathy biomarkers at diagnosis in relevant subgroups.

	BNP	*p*-Value	Percent Maximum VO_2_ (%)	*p*-Value	LGE %	*p*-Value
**Sex**		0.03		0.06		0.001
Male	221 (319)		74.1 (21.0)		11.1 (9.7)	
Female	389 (531)		82.6 (22.8)		5.6 (7.5)	
**Ethnicity**		0.94		0.02		0.08
White	293 (390)		81.3 (22.1)		7.1 (7.6)	
Black	285 (570)		66.4 (21.3)		12.3 (12.9)	
Other	361 (319)		66.4 (13.7)		6.8 (2.9)	
**Obstructive**		0.84		0.95		0.002
Yes	309 (491)		78.0 (23.4)		5.8 (7.4)	
No	295 (360)		78.3 (20.1)		11.3 (9.9)	
**Septal Phenotype**		0.11		0.05		0.004
Basal septal	226 (472)		82.6 (24.8)		5.1 (6.8)	
Reverse Curvature	401 (472)		72.0 (18.9)		11.2 (9.7)	
Apical	253 (272)		80.0 (22.6)		8.6 (9.9)	
Neutral	143 (147)		93.4 (16.9)		2.9 (4.7)	
**Genetic Test Result**		0.20		0.72		0.28
Pathogenic or likely pathogenic variant	394 (450)		75.3 (19.7)		10.9 (8.4)	
VUS	290 (476)		80.1 (23.0)		7.6 (8.9)	
Negative	226 (263)		78.3 (25.2)		7.8 (10.0)	

Lead time is calculated as (date of clinician diagnosis) minus (date of AI-identified ECG), with a positive lead time representing AI identification prior to clinician diagnosis. Data are reported as mean (SD). VUS = variant(s) of uncertain significance.

## Data Availability

The raw data supporting the conclusions of this article will be made available by the authors on request.

## Abbreviations

The following abbreviations are used in this manuscript:

AI	Artificial intelligence
ECG	Electrocardiogram
AI-ECG	AI-enabled ECG
DNN	Deep neural network
HCM	Hypertrophic Cardiomyopathy
